# Fatal Mesenteric Ischemia From a Presumed Paradoxical Embolism Through a Patent Foramen Ovale

**DOI:** 10.7759/cureus.102440

**Published:** 2026-01-27

**Authors:** George K Annan, Brice Njobe, Neaam I Al Bahadili, Orlando Palmer, Patrick O Berchie

**Affiliations:** 1 Internal Medicine, Piedmont Athens Regional Medical Center, Athens, USA

**Keywords:** acute mesenteric ischemia, anticoagulation interruption, bowel infarction, hypercoagulable state, paradoxical embolism, patent foramen ovale (pfo), renal cell carcinoma, superior mesenteric artery thrombosis, systemic embolism, venous thromboembolism (vte)

## Abstract

Acute mesenteric ischemia is a highly lethal vascular emergency most often caused by arterial embolism. Paradoxical embolism through a patent foramen ovale (PFO) is a well-recognized cause of cryptogenic stroke, but it is a rare and underrecognized cause of mesenteric arterial occlusion. A 68-year-old woman with a history of paradoxical embolism, chronic deep vein thrombosis, and a patent foramen ovale presented with sudden, severe abdominal pain and vomiting. She had previously undergone brachial artery embolectomy and had been maintained on long-term apixaban, which she later discontinued after being lost to follow-up. Computed tomography angiography revealed abrupt occlusion of the superior mesenteric artery and a newly identified left renal mass suspicious for malignancy. Telemetry showed no atrial fibrillation, blood cultures were negative, and no intracardiac thrombus was identified. Lower extremity Doppler ultrasound demonstrated chronic but not acute deep vein thrombosis. In the absence of alternative arterial embolic sources and given the temporal relationship to anticoagulation interruption, presumed paradoxical embolism was considered the most plausible mechanism. Despite emergent surgery, the patient developed extensive bowel necrosis and died after transition to comfort-focused care. This case adds to the limited literature on extracerebral paradoxical embolism and highlights the importance of sustained anticoagulation and longitudinal follow-up in patients with PFO and venous thromboembolism, particularly when additional hypercoagulable conditions are suspected.

## Introduction

Acute mesenteric ischemia is a vascular emergency characterized by sudden interruption of intestinal blood flow, most commonly due to arterial embolism or in situ thrombosis, and is associated with mortality exceeding 50% when bowel necrosis develops [1]. Typical clinical features include abrupt abdominal pain, nausea, vomiting, and rapid progression to bowel infarction if perfusion is not restored [1]. Arterial emboli, usually originating from atrial fibrillation or structural heart disease, account for a substantial proportion of cases. At the same time, malignancy and other hypercoagulable states increase the risk of both venous and arterial thrombosis [1-3]. Clinical presentation is often nonspecific, and early imaging findings may be subtle, contributing to delayed diagnosis and poor outcomes.

Paradoxical embolism occurs when thrombotic or embolic material originating in the venous system enters the systemic arterial circulation through a right-to-left intracardiac shunt, thereby circumventing pulmonary filtration. Patent foramen ovale (PFO) is present in approximately 25% of the general adult population but in about 50% of patients with cryptogenic ischemic stroke, suggesting its association with cryptogenic ischemic stroke through paradoxical embolism [4]. Although paradoxical emboli can involve systemic arterial circulation, reports of mesenteric arterial involvement remain uncommon and are largely limited to isolated case reports [5]. Diagnosis is particularly challenging because mesenteric ischemia is more often attributed to atrial fibrillation, atherosclerotic disease, or malignancy-associated arterial thrombosis, and extracerebral manifestations of paradoxical embolism are rarely considered in the absence of neurologic symptoms [1-3].

In hypercoagulable malignancy states, arterial ischemia often involves multiple mechanisms, with both embolic and in situ thrombotic processes contributing, depending on the clinical context. The coexistence of venous thromboembolism and an intracardiac shunt should prompt consideration of an embolic mechanism rather than primary arterial thrombosis. This distinction has important implications for diagnostic evaluation and management.

We report a fatal case of acute superior mesenteric artery thrombosis in a patient with a previously documented PFO and unprovoked venous thromboembolism who discontinued long-term anticoagulation and was later found to have a renal mass suspicious for malignancy, creating a uniquely high-risk substrate for paradoxical embolization. This report aimed to describe a rare extracerebral presentation of paradoxical embolism resulting in catastrophic mesenteric ischemia and to highlight the importance of sustained anticoagulation and longitudinal follow-up in patients with PFO and venous thromboembolism.

## Case presentation

A 68-year-old woman presented with the abrupt onset of severe abdominal pain and vomiting of a day's duration. Approximately four years earlier, she was admitted with acute left upper extremity ischemia manifested by numbness and loss of arterial flow. Arterial duplex ultrasound demonstrated acute occlusion of the left brachial, radial, and ulnar arteries, and she underwent emergent surgical embolectomy with restoration of distal pulses. On that same admission, lower extremity ultrasound revealed a left lower extremity deep vein thrombosis, establishing the coexistence of venous and arterial thrombosis.

Due to concerns for paradoxical embolus, a transthoracic echocardiography with agitated saline was obtained but was unrevealing; a transesophageal echocardiography demonstrated a patent foramen ovale, confirming a mechanism for paradoxical embolism (Video 1). No intracardiac thrombus, valvular disease, or aortic pathology was identified. A 30-day cardiac event monitor did not show atrial fibrillation. Hypercoagulable testing on hematology follow-up was negative, and the event was considered unprovoked at the time. She was discharged on apixaban with plans for indefinite anticoagulation. Cardiology documented that PFO closure was deferred because she was therapeutically anticoagulated and therefore protected against paradoxical embolism; closure was to be reconsidered only if anticoagulation was discontinued or contraindicated. The patient was subsequently lost to follow-up, and she had been off apixaban for about three years.

**Video 1:** Transesophageal echocardiogram demonstrating patent foramen ovale. Transesophageal echocardiographic saline contrast study showing immediate appearance of microbubbles in the left atrium following opacification of the right atrium, consistent with a right-to-left interatrial shunt through a patent foramen ovale. No left atrial appendage thrombus, valvular abnormality, or aortic atheroma was identified on transesophageal imaging.

**Figure 1:**
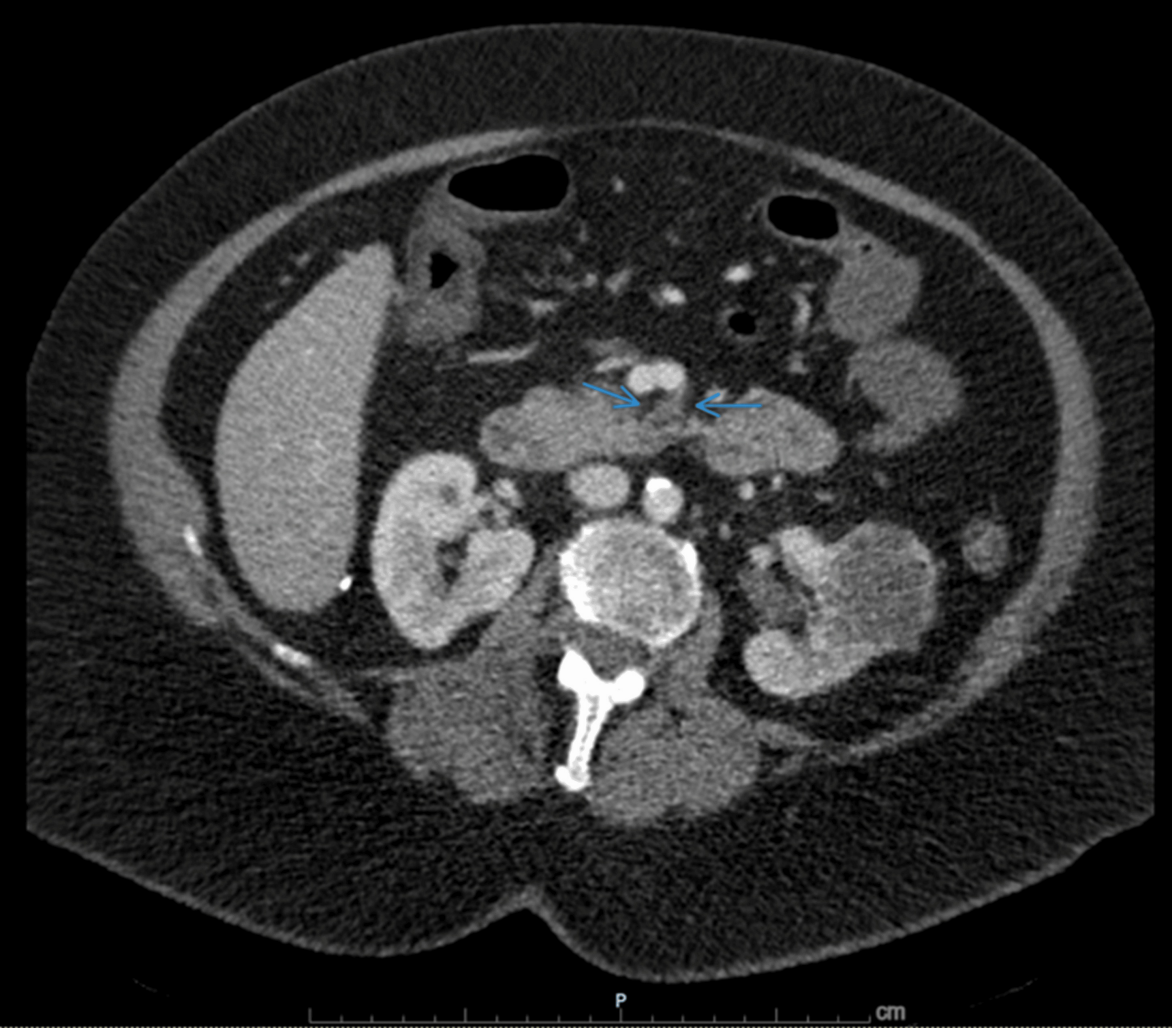
CT angiography demonstrating superior mesenteric artery occlusion. Contrast-enhanced CT of the abdomen demonstrating abrupt nonopacification of the superior mesenteric artery (arrows), consistent with acute arterial occlusion. At the time of imaging, no definite CT evidence of bowel ischemia was present.

**Figure 2:**
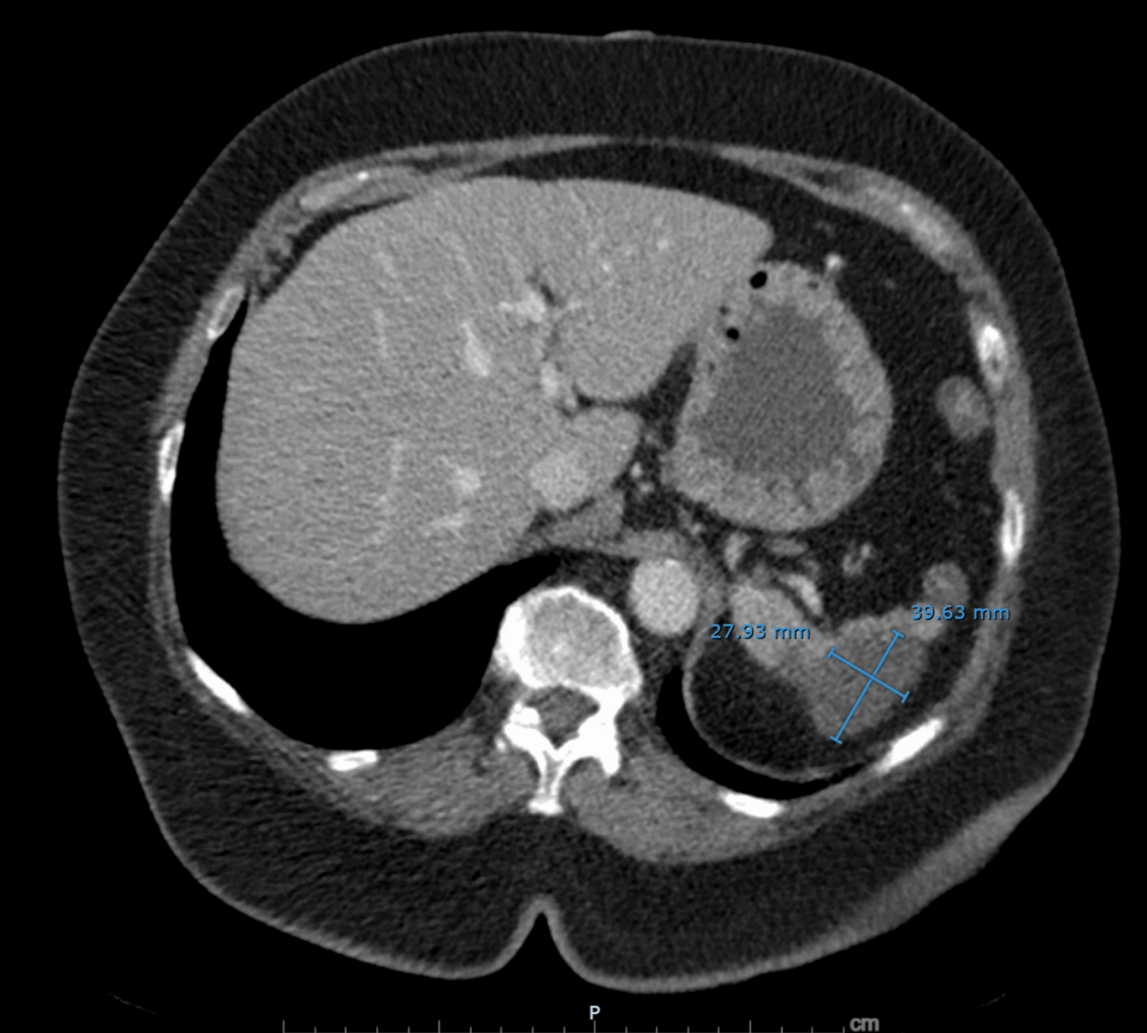
CT demonstrating abnormal splenic enhancement suspicious for splenic infarction. Contrast-enhanced CT of the abdomen demonstrates an atrophic, lobulated spleen with a new 4.0×2.8 cm low-density cystic lesion at the splenic dome and poor opacification of the splenic artery, findings favored to represent splenic infarction. A splenic metastasis was considered less likely.

**Figure 3:**
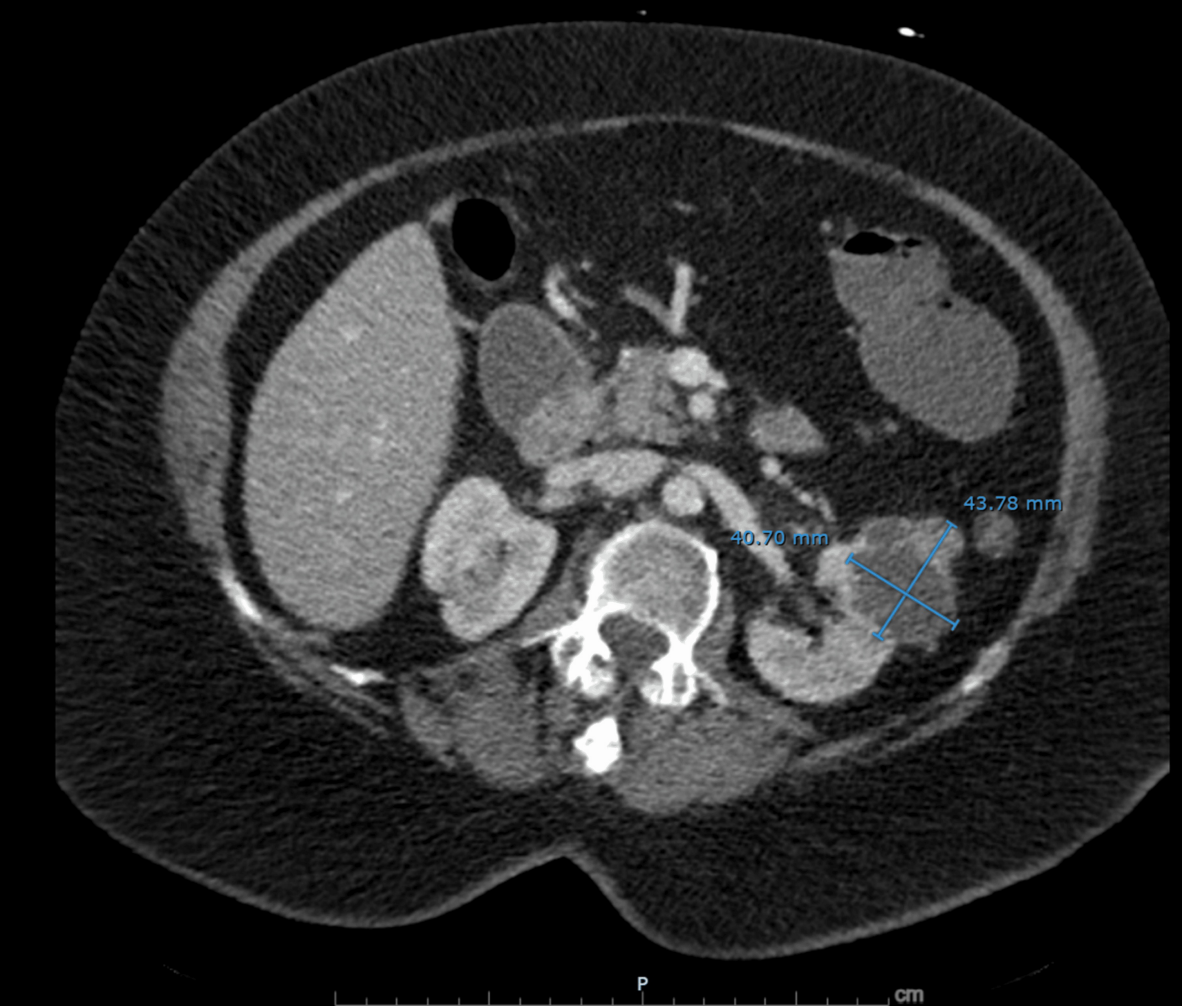
CT demonstrating a left renal mass suspicious for malignancy. Contrast-enhanced computed tomography of the abdomen demonstrates a heterogeneously enhancing exophytic mass in the left kidney measuring 4.4×4.1 cm. The mass does not appear to involve the left renal vein and is highly suspicious for renal cell carcinoma.

On presentation, vital signs were notable for hypertension and tachycardia, with a blood pressure of 180/84 mmHg, heart rate of 110 beats per minute, temperature of 98.1°F (36.7°C), respiratory rate of 16 breaths per minute, and oxygen saturation of 98% on room air. Initial differential diagnoses included acute pancreatitis, bowel obstruction, hollow viscus perforation, and aortic dissection. Computed tomography angiography of the chest, abdomen, and pelvis ruled out aortic dissection, aneurysm, and pancreatitis but demonstrated abrupt nonopacification of the superior mesenteric artery, abnormal splenic enhancement concerning for splenic infarction, and a 4.4 cm heterogeneously enhancing exophytic left renal mass highly suspicious for renal cell carcinoma (Figures 1-3).

**Figure 4:**
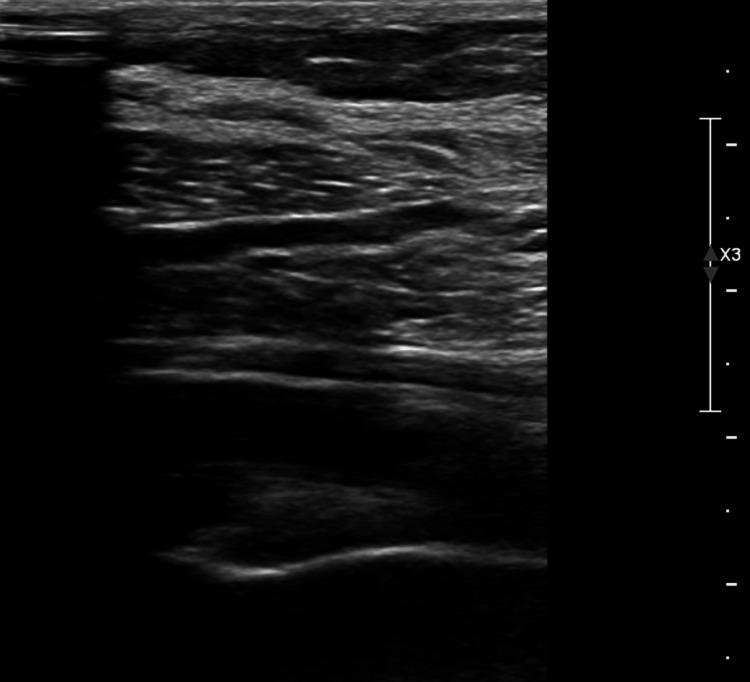
Lower extremity venous Doppler ultrasound demonstrating chronic deep vein thrombosis. The ultrasound shows chronic deep vein thrombosis with contracted vein walls involving the left proximal gastrocnemius vein, with no evidence of acute deep venous thrombosis.

Electrocardiography and continuous telemetry revealed no atrial fibrillation. The left lower extremity venous Doppler demonstrated chronic deep vein thrombosis without evidence of acute thrombosis (Figure 4). Emergent exploratory laparotomy was performed due to concern for evolving mesenteric ischemia. Ischemic small bowel was resected intraoperatively. On re-exploration 48 h later, more than half of the remaining small bowel was necrotic and nonviable, and further resection would have resulted in near-total bowel loss. After discussions with the family, care was transitioned to comfort measures, and the patient died several days later. No histopathologic confirmation of embolic material was obtained, and the renal mass was not biopsied; thus, malignancy was suspected rather than confirmed.

## Discussion

This case illustrates a rare but catastrophic manifestation of paradoxical embolism resulting in acute superior mesenteric artery occlusion and extensive bowel necrosis. The clinical significance lies not only in the unusual vascular territory involved but also in the fact that this event occurred in a patient with a previously recognized paradoxical embolic syndrome who became vulnerable after discontinuation of anticoagulation. The concurrence of a patent foramen ovale (PFO), chronic venous thrombosis, and a suspected hypercoagulable condition created a uniquely high-risk substrate for systemic embolization.

Paradoxical embolism is primarily linked to cryptogenic ischemic stroke, with PFO closure widely researched in this area [4]. To the best of our knowledge, extracerebral arterial embolization via patent foramen ovale, particularly involving the mesenteric circulation, has been described only sporadically in the literature, primarily in isolated case reports [5]. This has been attributed to factors such as preferential anatomic flow patterns and clinical recognition bias [4,6,7]. Our case is consistent with these prior observations but is distinguished by the presence of a previously documented paradoxical embolic event and a preventable fatal recurrence following interruption of therapy. Unlike previous reports that describe paradoxical embolism as a first presentation, this case uniquely documents the consequences of loss of protection in a patient known to have paradoxical embolic disease.

In situ thrombosis of the superior mesenteric artery may occur in the setting of advanced atherosclerosis or malignancy-associated hypercoagulability; however, several features in this case favored an embolic mechanism. Imaging demonstrated abrupt nonopacification of the proximal superior mesenteric artery without evidence of chronic arterial narrowing, calcification, or atherosclerotic disease. Additionally, radiographic findings favored splenic infarction in a second arterial territory, supporting systemic embolization rather than isolated local thrombosis. Alternative arterial embolic sources were systematically evaluated and excluded. Continuous telemetry and electrocardiography revealed no atrial fibrillation or other arrhythmia. Transesophageal echocardiography demonstrated no intracardiac thrombus, valvular pathology, or left atrial appendage thrombus, and imaging showed no evidence of aortic atheroma or mural thrombus. In the setting of chronic venous thromboembolism and a documented PFO, paradoxical embolism was therefore considered the most plausible etiology.

Although the left renal mass was radiographically highly suspicious for malignancy, no histopathologic confirmation was obtained. Accordingly, malignancy was considered a suspected contributor to a hypercoagulable state rather than a confirmed etiologic factor. Nevertheless, the presence of a large renal mass in conjunction with recurrent thromboembolic events raised concern for malignancy-associated thrombosis as an amplifying risk factor for embolic events. From a clinical perspective, this case underscores several important management principles. First, in patients with unexplained arterial thrombosis, particularly outside the cerebral circulation, clinicians should consider paradoxical embolism and pursue evaluation for intracardiac shunts. Second, patients with PFO and venous thromboembolism require sustained anticoagulation and longitudinal follow-up. Third, hypercoagulable conditions may significantly amplify thrombotic risk and lower the threshold for recurrent embolic events when anticoagulation is interrupted.

Earlier resumption of anticoagulation or reassessment for PFO closure may have mitigated embolic risk; however, PFO closure had previously been deferred because the patient was managed with long-term therapeutic anticoagulation, which is generally considered protective against paradoxical embolism. Closure was to be reconsidered only if anticoagulation was discontinued or contraindicated; however, the patient was subsequently lost to follow-up, and anticoagulation was discontinued without reassessment of PFO management.

Important knowledge gaps remain regarding the optimal management of patients with PFO who experience extracerebral paradoxical embolism. Unlike cryptogenic stroke, evidence for PFO closure in noncerebral systemic embolism is minimal. The Society for Cardiovascular Angiography and Interventions (SCAI) guidelines conditionally recommend PFO closure over medical therapy alone in individuals with systemic embolism (after excluding other potential embolic etiologies), but this recommendation is based on very low certainty evidence [8]. The Patent Foramen Ovale and Cryptogenic Embolism (PC) trial included patients with peripheral thromboembolic events alongside stroke patients, but this heterogeneous population made interpretation difficult, and the study was also underpowered [9-11]. In clinical practice, this uncertainty necessitates individualized, multidisciplinary decision-making that weighs embolic recurrence risk, feasibility of sustained anticoagulation, bleeding risk, comorbid conditions, and patient adherence and follow-up reliability.

Additionally, the interaction between suspected malignancy-associated hypercoagulability and intracardiac shunts is poorly studied [4,6]. Future randomized controlled trials and registry data may help clarify which patients are at the highest risk and whether alternative strategies, such as intensified surveillance, earlier closure, or prolonged anticoagulation, are warranted. This case also highlights the need for structured long-term surveillance in patients with known PFO and venous thromboembolism. Regular reassessment of anticoagulation adherence, evaluation for evolving hypercoagulable conditions, and reconsideration of PFO management when protective therapy is interrupted may help prevent catastrophic extracerebral embolic events in high-risk patients.

This report has several limitations. As a single case, causality cannot be definitively proven. Although the presence of PFO, venous thrombosis, and systemic embolization strongly supports paradoxical embolism, direct visualization of thrombus transit is not possible. The Risk of Paradoxical Embolism (RoPE) score and PFO-Associated Stroke Causal Likelihood (PASCAL) classification systems can help estimate the likelihood that a PFO is causally related to an embolic event, but these tools were developed and validated specifically for cerebrovascular events, not for extracerebral embolism [12,13].

## Conclusions

This case highlights a rare and fatal extracerebral manifestation of paradoxical embolism resulting in acute mesenteric ischemia in a patient with a known patent foramen ovale and prior venous thromboembolism. It underscores the critical importance of sustained anticoagulation adherence and longitudinal follow-up in high-risk patients, particularly when hypercoagulable conditions, such as suspected malignancy, are present. Clinicians should consider paradoxical embolism in cases of unexplained arterial ischemia outside the cerebral circulation and ensure timely reassessment of embolic risk when anticoagulation is interrupted or follow-up is lost. These observations are hypothesis-generating rather than practice-changing, highlighting the need for prospective studies to better define optimal management strategies for extracerebral paradoxical embolism.
